# Synthesis of Atomically Thin h-BN Layers Using BCl_3_ and NH_3_ by Sequential-Pulsed Chemical Vapor Deposition on Cu Foil

**DOI:** 10.3390/nano12010080

**Published:** 2021-12-29

**Authors:** Hongseok Oh, Gyu-Chul Yi

**Affiliations:** 1 Department of Physics and Integrative Institute of Basic Science, Soongsil University, Seoul 06978, Korea; hoh@ssu.ac.kr; 2 Department of Physics and Astronomy and Institute of Applied Physics, Seoul National University, Seoul 08826, Korea

**Keywords:** h-BN, BCl_3_, NH_3_, sequential-pulsed CVD

## Abstract

The chemical vapor deposition of hexagonal boron nitride layers from BCl_3_ and NH_3_ is highly beneficial for scalable synthesis with high controllability, yet multiple challenges such as corrosive reaction or by-product formation have hindered its successful demonstration. Here, we report the synthesis of polycrystalline hexagonal boron nitride (h-BN) layers on copper foil using BCl_3_ and NH_3_. The sequential pulse injection of precursors leads to the formation of atomically thin h-BN layers with a polycrystalline structure. The relationship between growth temperature and crystallinity of the h-BN film is investigated using transmission electron microscopy and Raman spectroscopy. Investigation on the initial growth mode achieved by the suppression of precursor supply revealed the formation of triangular domains and existence of preferred crystal orientations. The possible growth mechanism of h-BN in this sequential-pulsed CVD is discussed.

## 1. Introduction

Hexagonal boron nitride (h-BN) is a versatile building block for emerging electronic and optoelectronic devices. For example, it is widely employed as scattering-free substrates, dielectric layers, or tunneling layers in two-dimensional (2-D) material-based electronics [1,2,3]. Another application includes templates for van der Waals epitaxy, photoactive channels for deep ultra-violet (UV) optoelectronic devices, single-photon emitters for quantum informatics, or recent artificially twisted Moiré layers [4,5,6,7,8,9,10,11]. Accordingly, large-area synthesis of h-BN becomes of high importance to realize its strong potential for novel electronics outside the laboratory. Up to date, various chemical vapor deposition (CVD) techniques has been developed to synthesize h-BN layers in a large scale [12,13,14,15].

Solid or liquid precursors have been widely used for the large-scale growth of h-BN [16]. However, from an applicational perspective, replacing those precursors with industrial gases can be highly advantageous for easy technology transfer to the manufacturing site [17]. Promising candidates are boron halide, chloride, or diborane as boron precursors, and NH_3_ as a nitrogen precursor [18]. These gases are commonly used in microelectronics industry, and they are suitable for scalable manufacturing. However, despite such advantages, the use of such gases for the synthesis of boron nitride (BN) mostly focused on nanoscale conformal coatings [19,20]. The synthesis of atomically thin, crystalline h-BN which can be transferred onto foreign substrates has been rarely reported.

Here, we report the synthesis of atomically thin h-BN films from BCl_3_ and NH_3_ precursors using sequential-pulsed chemical vapor deposition on Cu foil and discuss their structural and physical characteristics. The use of catalytic substrate under high-growth temperature led to the formation polycrystalline h-BN over the large area. Precise control over precursor injection minimized the by-product formation of BCl_3_ and NH_3_ precursors. Discussions for the physical and structural properties of the synthesized film, along with the investigation of initial growth mode, is followed.

## 2. Materials and Methods

### 2.1. Growth of h-BN Layers

The sequential-pulsed cold-wall CVD was carried out using a homemade custom vertical CVD chamber. The graphite susceptor was remotely heated by radiofrequency (RF) heater. The Cu foil was cleaned by sonicating in solvent using acetone and IPA; then, it was placed on the graphite susceptor. High-purity N_2_ and H_2_ were used as ambient gases. The flow rate of N_2_ and H_2_ was set to 1000 sccm for each gas. Before the growth, the susceptor was heated to the target temperature (800–1000 °C) at the rate of 100 °C/min and stabilized for 5 min. For the growth of h-BN, high-purity BCl_3_ and NH_3_ were used. The flowrate of BCl_3_ ranged for 3–5 sccm, and for NH_3_, it ranged 200–500 sccm. The injection and purge time varied from 5 to 10 s depending on the growth. After the growth was done, the susceptor was cooled down naturally under the flow ambient gases.

In specific, to study the effect of growth temperature on h-BN, the flow rate of each gas was 1000/1000/5/200 sccm for H_2_/N_2_/BCl_3_/NH_3_, and the injection time and purging time were 5/5/5/5 s (BCl_3_/purge/NH_3_/purge). The total number of cycles was 15, and the growth temperature ranged from 800 to 1000 °C. For the observation of triangular domain formation, the flow rate of each gas was 1000/1000/5/200 for H_2_/N_2_/BCl_3_/NH_3_, and the injection time and purging time were 10/20/10/20 s (BCl_3_/purge/NH_3_/purge). The total number of cycles was 10 and the growth temperature was 1000 °C.

### 2.2. Transfer of h-BN Layers

Poly(methyl methacrylate) (PMMA) was spun-coated on the h-BN grown Cu foil. The spin speed was 1000 rpm. Then, the Cu foil was placed on the hot plate at the temperature of 150 °C to bake the PMMA layer. Before etching the Cu foil, the back side of the Cu foil was treated by oxygen plasma (50 W, 5 min) to remove the unwanted h-BN residues. Ammonium persulfate solution was used to slowly etch the Cu foil. The typical etch time ranged from a few hours to a day. The remaining PMMA/h-BN layer was rinsed several times by DI water; then, it was transferred onto the target substrate (SiO_2_ or TEM grid). After drying in ambient condition, the PMMA layer was removed in acetone for a few hours. The sample was finally rinsed using methanol to avoid the formation of residues.

### 2.3. Characterization

For the FE-SEM imaging, AURIGA (Carl Zeiss Microscopy Deutschland GmbH, Oberkochen, Germany) was used. For the TEM study, we used Tecnai F20 (Materials & Structural Analysis Division (MSD), Thermo Fisher Scientific, Hillsboro, OR, USA) and JEM-2100F (JEOL Ltd., Akishima, Tokyo, Japan).

## 3. Results

### 3.1. A Growth Setup for Sequential-Pulsed CVD

**Figure 1:**
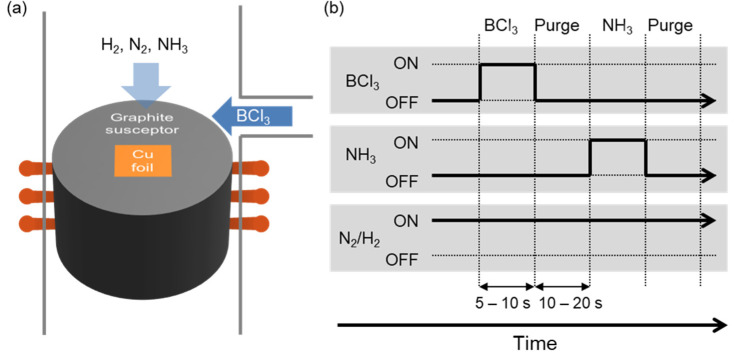
Synthesis of the h-BN layers using sequential-pulsed CVD process. (**a**) Schematic illustration of the cold-wall CVD system. (**b**) Diagram for the injection sequence of the gas precursors (BCl_3_ and NH_3_) and ambient gases (N_2_ and H_2_).

The synthesis of h-BN was carried out by the cold-wall CVD system, as schematically illustrated in Figure 1a. Here, the susceptor is remotely heated by the radiofrequency (RF) coil, while the chamber wall is kept cold. This cold-wall system allows chemical reactions to take place only on top of the susceptor and suppresses pre-reaction or outgassing from the chamber. Therefore, the generation of contaminants is minimized, and high-purity growth is achieved. For the growth of h-BN, a piece of copper foil served as a catalytic substrate. A mixture of N_2_ and H_2_ was used as an ambient gas, and BCl_3_ and NH_3_ were employed as precursors for boron and nitrogen, respectively. Especially, to avoid the formation of ammonium chloride, which is an unwanted by-product of BCl_3_ with NH_3_, the reaction gases were introduced through two different paths. Ambient gases (N_2_, H_2_) and NH_3_ were injected from the showerhead at the top, while BCl_3_ was fed from the narrow branch attached at the side of the chamber. It is noteworthy that the copper foil was partially covered with a sapphire wafer to investigate the effect of suppressing the precursor supply, which will be discussed in the next section.

We employed an ‘injection and purge’ strategy to control the growth dynamics and to avoid unwanted by-products, which was inspired from the ALD process [21]. The sequence of introducing gas precursors is described in Figure 1b. In each cycle, BCl_3_ is first introduced for 5–10 s followed by 10–20 s of purging process (i.e., no injection of precursors); then, NH_3_ is introduced for 5–10 s followed by the same 10–20 s of purging process. The specific time for precursor injection and purging varied by the growth. Generally, to achieve a fully merged h-BN film, the total growth time was 5–10 min or 10–15 cycles. It should be noted that without the sequential pulse injection technique, turbostatic structures and a dense white solid by-product of ammonium chloride (NH_4_Cl) are formed from the reaction of BCl_3_ and NH_3_. Additionally, in such a condition, the growth rate become too fast to yield atomically thin h-BN layers.

### 3.2. Synthesis of h-BN Layers and Their Physical Characteristics

**Figure 2:**
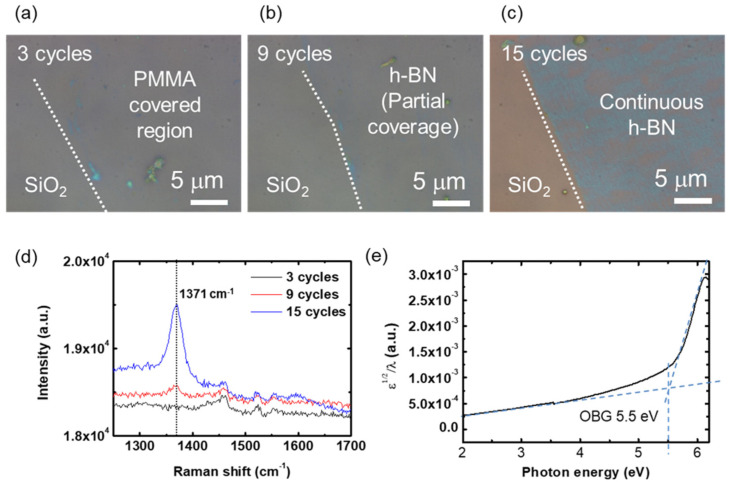
Synthesis of h-BN layers and its physical properties. (**a**–**c**) Optical microscope images of the transferred h-BN films on SiO_2_/Si substrates grown for (**a**) 3 cycles, (**b**) 9 cycles, and (**c**) 15 cycles. (**d**) Raman spectra of the h-BN films in (**a**–**c**). (**e**) Tauc’s plot of the UV-Vis spectrum of the synthesized h-BN film.

H-BN films were grown on the Cu foil with different numbers of cycles and transferred onto SiO_2_/Si for further characterization. A typical wet transfer method was used to transfer the grown h-BN. Detailed procedures about the growth and transfer are described in the Materials and Method section. Figure 2a–c show the optical microscope images of the transferred films. It was difficult to find any optical contrast between SiO_2_ and the poly(methyl methacrylate) (PMMA) covered region for the 3-cycle grown sample, indicating that the number of cycles was not enough. When it increased to nine cycles, the transferred film exhibited a little contrast difference to the bare SiO_2_ region (Figure 2b). Still, it is difficult to draw the border line between h-BN and bare SiO_2_. For the sample grown over 15 cycles, a clear contrast difference was observed between the h-BN covered region and bare SiO_2_ region, as shown in Figure 2c. The trend indicates that to achieve a continuous film, at least 15 cycles are required. From the high-resolution (HR) TEM images of the sample grown over 15 cycles in Figure A2, which depicts cross-sectional structures from accidentally folded regions, the number of layers in this film is expected to be ≈4 or higher. Interestingly, the contrast was not uniform, and thicker regions (brighter region) are distributed along the single direction, indicating that nucleation mainly took place along the wrinkle of the Cu foil. Further substrate engineering such as electropolishing will lead to higher uniformity.

Raman spectrum was taken for these samples. No signal was observed from three-cycle grown samples, but a weak peak appeared at 1371 cm^−1^ from the nine-cycle grown sample. A strong peak appeared at the same position from the sample grown by 15 cycles. These peaks correspond to the characteristic E_2g_ peak of h-BN, which evidences the formation of h-BN crystal. An optical band gap was extracted from the grown h-BN film. For this, UV-Vis spectrum was taken, and from the spectrum, Tauc’s plot or $\varepsilon^{\frac{1}{2}}/\lambda$ vs. hv plot was calculated, as shown in Figure 2e. An optical band gap (OBG) of 5.5 eV was extracted from the extrapolation of baseline and peak absorption near 6 eV, which matches well with typical OBG values of CVD h-BN.

### 3.3. Effect of Growth Temperature on Crystallinity

**Figure 3:**
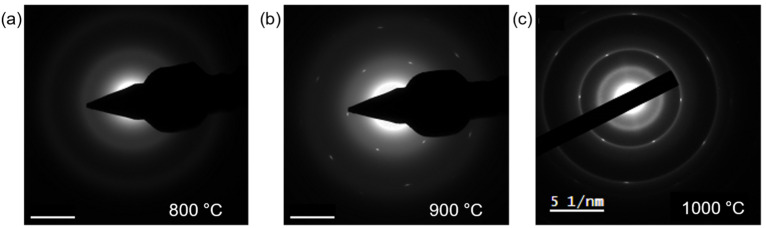
Relationship between growth temperature and crystallinity. (**a**–**c**) SAED patterns of the h-BN films synthesized at different temperatures, (**a**) 800 °C, (**b**) 900 °C, and (**c**) 1000 °C, respectively. Scale bars: 5 1/nm.

**Figure A1:**
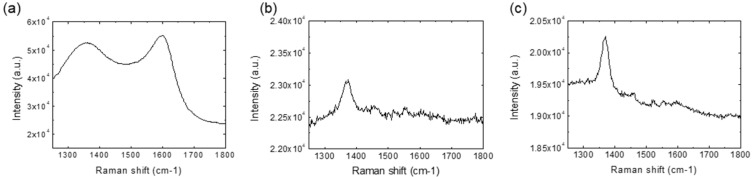
Raman spectrum of the synthesized h-BN at different temperatures: (**a**) 800 °C, (**b**) 900 °C, and (**c**) 1000 °C.

**Figure A2:**
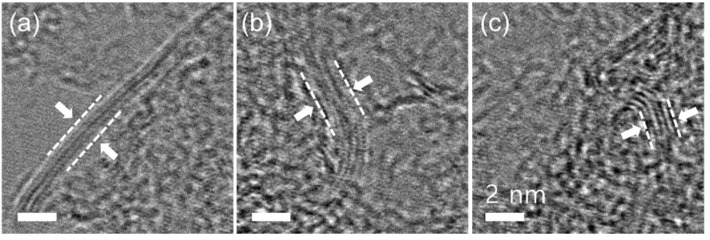
HR-TEM images of h-BN films with accidentally folded regions, captured from different spots. The film was grown at 1000 °C for 15 cycles. Approximate numbers of layers are (**a**) 4 layers, (**b**) 5 layers, and (**c**) 5 layers. Scale bar: 2 nm.

We further investigated the effect of growth temperature on the crystal structure of the grown h-BN. Specific growth parameters are described in the experimental section. Continuous h-BN atomic layers with a negligible amount of turbostatic structures were achieved even without suppressing precursor supply (i.e., no sapphire wafer was used) when the injection times of BCl_3_ and NH_3_ were reduced 5/5 sec for BCl_3_/NH_3_, respectively. We took SAED patterns and Raman spectrums for the h-BN films grown under different temperatures. At 800 °C, no hexagonal spots were observed in the SAED pattern (Figure 3a), meaning that there existed no hexagonal lattices. The corresponding Raman spectrum (Figure A1a) indicates that the film was mostly composed of amorphous carbon. When the growth temperature was increased to 900 °C, we observed clear hexagonal spots in SAED, indicating a perfect hexagonal lattice structure (Figure 3b), at least in the region with an aperture size of 500 nm. The corresponding Raman spectrum (Figure A1b) exhibited a characteristic peak of h-BN near 1366 cm^−1^, which is coming from the stretch and compression of h-BN hexagon. At a further elaborated temperature of 1000 °C, a ring pattern appeared along with hexagonal spots in the SAED pattern (Figure 3c). This indicates that randomly oriented h-BN nanocrystals were co-existing with aligned h-BN domains in the film. On the other hand, a stronger h-BN E_2g_ peak with smaller FWHM was observed in the corresponding Raman spectrum (Figure A1c) for the 1000 °C grown h-BN film. The study suggests that while enough thermal energy should be provided to form a hexagonal lattice structure, precise control of the precursor feed rate and substrate temperature and growth temperature is required to avoid the formation of randomly oriented domains.

### 3.4. Initial Growth Mode and Preferred Crystal Orientation

We observed the formation of triangular domains, which is the characteristic feature of the CVD h-BN growth [22]. It happened especially in the region a few millimeters inside the edge of the covering sapphire wafer, where precursors should travel the narrow gap between the Cu foil and the sapphire wafer to arrive. Since the amount of delivered precursors becomes smaller as the distance from the edge of the wafer becomes longer, it is reasonable that the formation of individual domains was the result of suppressed precursor supply. The observation of individual domains under suppressed precursor supply was reported in many research studies regarding graphene and h-BN [23,24].

**Figure 4:**
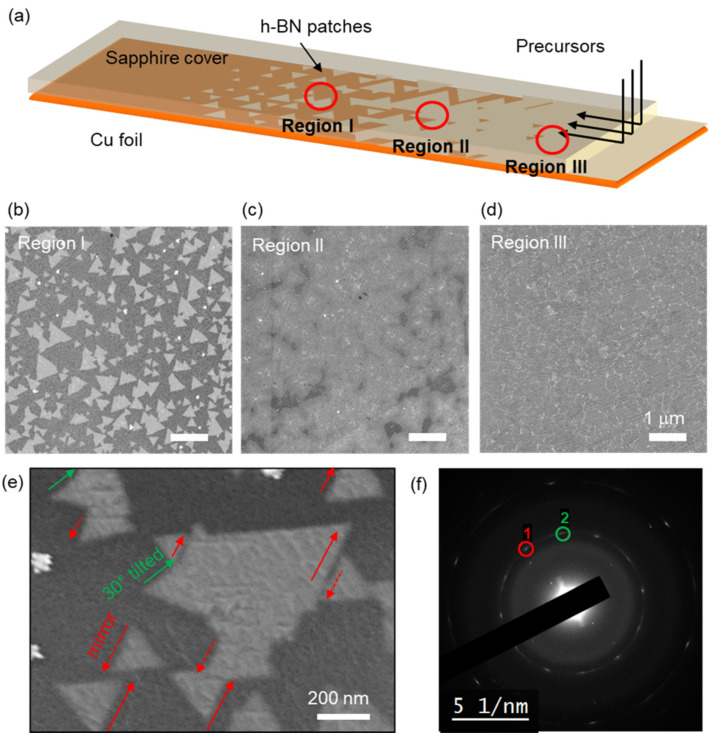
Observation of triangular patches of h-BN grown under minimized feeding rate using sapphire wafer as a cover. (**a**) Illustration of the experimental set-up and growth behavior of h-BN microdomains on Cu foil. (**b**–**d**) FE-SEM images taken from different location of the region under the sapphire cover. (**b**), (**c**), and (**d**) correspond to the regions I, II, and III marked by red circles in (**a**), respectively. Alignment of the crystal orientations of h-BN triangular nanopatches. (**e**) FE-SEM image of h-BN nanopatches synthesized on Cu foil. Most h-BN patches were aligned to a single direction (red arrows), or its mirror (red-dashed arrows), or tilted by 30° (green arrows). (**f**) Typical SAED pattern of the synthesized h-BN film. Two major domain orientations separated by 30° are marked as 1 and 2, respectively.

As illustrated in Figure 4a, under the region covered by the sapphire wafer, h-BN triangular nano patches, a partially merged layer, and a full coalescence film were identified depending on the distance from the covering wafer. The specific growth parameters are described in experimental section. Upon the region which was about 5 mm inside from the edge, multiple triangular patches with size ranging from 0.1 to 1 mm were identified, as clearly seen in the FE-SEM image of Figure 4b. The triangular shape is commonly observed from other research on h-BN synthesis using a metallic substrate, because edges with nitrogen termination are more energetically favorable [15]. On the other hand, a layer with increased coverage is observed on the region closer to the edge, i.e., about 2 mm inside from the edge, as shown on Figure 4c, due to the increased dose to the precursor gases. Lastly, fully coalescence film was observed near the edge of the wafer, as depicted by Figure 4d, due to the further increase in the precursor supply. These results suggest that suppressing the precursor injection in a controllable way, i.e., the dilution of precursors, can help precisely control the synthesis of h-BN atomic layers, such as nucleation density, control of domain size, or the number of layers without use of covering wafers.

Interestingly, the triangular h-BN domains exhibited preferred crystal orientations. The FE-SEM image of Figure 4e shows the typical domains observed in the growth. Triangular domains are mostly oriented to the single direction, which is indicated by red arrows. At the same time, orientations of multiple triangles are rotated by 180° (red-dashed arrows), which corresponds to the mirror domains. There also existed tilted domains (green arrows) whose orientation is rotated by 30° from the red arrow. From this observation, if we set 0° as the direction of the red arrow, 0° and 30° appears to be the two specific preferred orientations, and 60° and 90° are another pair of preferred orientations which are mirror domains to 0° and 30° domains.

To quantitatively analyze the spatial distribution of crystal orientations, transmittance electron microscopy (TEM) study was followed. Figure 4f shows the selective area electron diffraction (SAED) pattern of the grown h-BN film, with an aperture size of 4.5 mm. A set of hexagonal spots corresponds to a lattice spacing of 2.2 Å, which well matched with the known interlayer distance of h-BN (100) plane [25]. Furthermore, two set of hexagonal spots were separated by 30°, which is consistent with the observation in FE-SEM image. Here, because the mirror domains exhibit same diffraction patterns, only two major directions can be distinguished by the diffraction.

In our study, polycrystalline Cu foil served as a substrate; hence, it is difficult to discuss the precise crystallographic relationship between h-BN and Cu. Nevertheless, the growth result suggests that by further investigating the relationship between grown h-BN domains and underlying Cu crystals and engineering the substrate to exhibit certain crystallographic direction, one can achieve atomically thin h-BN films with single or controlled domains using this sequential-pulsed CVD method.

## 4. Discussion

The growth of BN from gas precursors was reported in various ways so far. In the early stage of the research, the CVD of BN was investigated in application to semiconductor device from precursor combinations of B_2_H_6_ + NH_3_, BCl_3_ + NH_3_, BF_3_ + NH_3_, BBr_3_ + NH_3_, etc. [18]. Nowadays, modern growth techniques such as atomic layer deposition (ALD) or a similar approach such as sequential injection CVD emerged as novel way to achieve better controllability over thickness, stoichiometric ratio, etc. [26]. Different growth strategies are summarized in Table 1. When non-catalytic ceramic substrates are employed, the resulting films were of amorphous or turbostatic structures, depending on the growth temperature [19,20,27,28,29,30]. These films were used in coating nanoparticles, fiber fabrics, or achieving an ultrasmooth substrate for novel 2D electronics. On the other hand, dosing precursors on single crystalline transition metal substrates resulted in mono- or multi-layer epitaxial h-BN, which is suitable for nanoelectronics applications [31,32,33,34]. However, the transfer of the grown epitaxial film should be followed to fully utilize the potential of the film.

Usually, large-scale atomically thin h-BN films were grown on transition metal substrates such as Cu or Ni foils using solid (ammonia–borane) or liquid (borane) precursors [16]. Over the years, innovative approaches have been cumulated such as use of single crystalline substrates with a non-symmetric facet for oriented nucleation, electrochemical delamination for damage-free transfer, or self-collimated growth on liquid substrate, resulting in wafer-scale, single-crystal monolayer h-BN films [35,36,37,38,39,40]. Therefore, revisiting the same path of technological progress using gas precursors is important to realize high-quality, large-scale h-BN atomic layers in a more scalable way. Low-pressure CVD of h-BN layers on Cu or Ni foil using B_2_H_6_ and NH_3_ is an example of this approach, but further efforts have been rarely reported. Our work presents a formation of h-BN using industrial gases, from nucleation stage to the continuous film, which is similar to the early-stage research of CVD h-BN from conventional solid or liquid precursors. Further engineering on substrate, precise control on temperature, flowrate, pressure, or gas mixture ratio will lead to high-quality atomic h-BN layers such as single domain growth, control on number of layers, or successful heterostructures with other 2D materials.

## 5. Conclusions

In conclusion, we synthesized atomically thin h-BN layers using BCl_3_ and NH_3_ precursors. The successful synthesis of atomically thin h-BN layers was achieved by the sequential injection of each precursor on a catalytic substrate at elaborated temperature. The initial growth mode was observed by suppressing the precursor supply, from which triangular domains were exhibited that were aligned to a few preferred orientations. Growth temperature study indicated that at too low growth temperature, h-BN crystal did not form, while too high growth temperature led to randomly oriented nano-sized domains. Further advances in crystallinity and growth uniformity are expected in the future by employing state-of-the-art techniques such as substrate engineering or the use of single crystal substrates. This research provides a general route to synthesize atomically thin h-BN layers based on industrial gases, leading to the scalable production of 2D insulating layers for future applications.

## Figures and Tables

**Table 1:** Synthesis of BN from industrial gas precursors.

Precursors	Growth Method	Substrate	Growth Temperature	Properties of the Resulting Film	Ref.
BBr_3_, NH_3_	Low-pressure, hot-wall ALD	Silica substrate	400–750 °C	Turbostatic BN, very smooth (surface roughness of 0.3–0.5 nm)	[28]
	Laser-assisted ALD		250–750 °C	Hydrogen-terminated turbostatic BN	[27]
	Hot-wall ALD	Silica, Si/SiN_x_, or Anodic aluminum oxide (AAO)	750 °C	BN nanoporous membrane (turbostatic BN)	[20]
BCl_3_, NH_3_	Chemical vapor infiltration (CVI)	SiC fiber fabric	Deposition at 843 °C Heat-treated at 1050 °C	Conformal BN coating with thickness ranging 545–745 nm, Excellent thermal stability	[29]
	ALD	ZrO_2_ particles	226.85 °C (500 K)	Conformal a-BN coating with thickness of ≈25 Å	[19]
	ALD using UHV chamber	Ru(0001)	2-cycle deposition at 276.85 °C (550 K) UHV anneal at 726.85 °C (1000 K)	$R30(\sqrt{3}\times \sqrt{3}$) BN(111) monolayer on Ru(0001)	[31]
	Atomic layer epitaxy (ALE) using UHV chamber	Co(0001)	326.85 °C (600 K)	Multi-layer h-BN(0001) films (up to seven layers)	[32]
	ALD using UHV chamber	Co(0001)	Deposition at 550 K Annealing at 700 K	Multi-layer h-BN films with azimuthal registry	[33]
	ALD using UHV chamber	RuO_2_ on Ru(0001)	Deposition at 326.85 °C (600 K) Annealing at 526.85 °C (800 K)	Multi-layer epitaxial h-BN(0001) films	[34]
	Hot-wall LPCVD	Si	900–1400 °C	Micrometer-thick turbostatic BN films with mixture of poorly and highly organized domains	[30]
	Cold-wall ALD	SiO_2_/Si	600 °C	Ultra-smooth nanocrystalline layered-BN thin film	[41]
	Sequential-pulsed CVD	Cu	900–1000 °C	Polycrystalline h-BN layers	This work
B_2_H_6_, NH_3_	LPCVD and sequential pulsed-CVD	Ni, Cu, or sapphire	1025 °C	Thin (1–5 layers) and thick (~100 layers) polycrystalline h-BN film	[17]
	Low-pressure (<10^6^ Torr) exposure	Ni	676.85 °C (950 K)	Self-limited monolayer BN	[42]

## Data Availability

The data presented in this study are available on reasonable request from the corresponding author.
